# Afadin Regulates Puncta Adherentia Junction Formation and Presynaptic Differentiation in Hippocampal Neurons

**DOI:** 10.1371/journal.pone.0089763

**Published:** 2014-02-27

**Authors:** Daisaku Toyoshima, Kenji Mandai, Tomohiko Maruo, Irwan Supriyanto, Hideru Togashi, Takahito Inoue, Masahiro Mori, Yoshimi Takai

**Affiliations:** 1 Department of Biochemistry and Molecular Biology, Kobe University Graduate School of Medicine, Kobe, Hyogo, Japan; 2 Faculty of Health Sciences, Kobe University Graduate School of Health Sciences, Kobe, Hyogo, Japan; 3 CREST, Japan Science and Technology Agency, Kobe, Hyogo, Japan; Virginia Tech Carilion Research Institute, United States of America

## Abstract

The formation and remodeling of mossy fiber-CA3 pyramidal cell synapses in the stratum lucidum of the hippocampus are implicated in the cellular basis of learning and memory. Afadin and its binding cell adhesion molecules, nectin-1 and nectin-3, together with N-cadherin, are concentrated at puncta adherentia junctions (PAJs) in these synapses. Here, we investigated the roles of afadin in PAJ formation and presynaptic differentiation in mossy fiber-CA3 pyramidal cell synapses. At these synapses in the mice in which the *afadin* gene was conditionally inactivated before synaptogenesis by using nestin-Cre mice, the immunofluorescence signals for the PAJ components, nectin-1, nectin-3 and N-cadherin, disappeared almost completely, while those for the presynaptic components, VGLUT1 and bassoon, were markedly decreased. In addition, these signals were significantly decreased in cultured afadin-deficient hippocampal neurons. Furthermore, the interevent interval of miniature excitatory postsynaptic currents was prolonged in the cultured afadin-deficient hippocampal neurons compared with control neurons, indicating that presynaptic functions were suppressed or a number of synapse was reduced in the afadin-deficient neurons. Analyses of presynaptic vesicle recycling and paired recordings revealed that the cultured afadin-deficient neurons showed impaired presynaptic functions. These results indicate that afadin regulates both PAJ formation and presynaptic differentiation in most mossy fiber-CA3 pyramidal cell synapses, while in a considerable population of these neurons, afadin regulates only PAJ formation but not presynaptic differentiation.

## Introduction

Synapses are specialized intercellular junctions that are indispensable for neuronal transmission. Most excitatory synapses are formed on the heads of dendritic spines and have asymmetric structures. Synapses contain at least two types of junctional structures: synaptic junctions (SJs) and puncta adherentia junctions (PAJs) [1]. PAJs resemble adherens junctions (AJs) of epithelial cells in their molecular architecture and are regarded as mechanical adhesion sites between axons and their target dendrites, while SJs function as sites of neurotransmission. SJs are associated both with synaptic vesicles docked at the presynaptic active zones where Ca^2+^ channels are localized, and with postsynaptic densities (PSDs) where specific neurotransmitter receptors are concentrated. PAJs, in contrast, contain symmetrical paramembranous dense materials and are not associated with synaptic vesicles. Both SJs and PAJs are highly developed as separate clusters consisting of distinctive macromolecular complexes in mossy fiber-CA3 pyramidal cell synapses in the stratum lucidum of the hippocampus. The synapses in this area undergo activity-dependent remodeling and reorganization, which is implicated in the cellular basis of learning and memory [2]. However, the molecular mechanisms underlying activity-dependent remodeling and reorganization are poorly understood. Moreover, it is not fully understood how contacts between axons and dendrites are initiated, or how presynaptic and postsynaptic components are recruited to the contact sites to establish synapses.

Many cell-cell adhesion molecules (CAMs) are localized at synapses and are implicated in synaptogenesis [3], [4]. Among them, N-cadherin was shown to be localized at the presynaptic and postsynaptic sides of PAJs, but not at SJs, in an adult cerebellar nucleus [5]. Moreover, neuroligin 1 and neuroligin 2 were shown to be localized at the PSDs of excitatory synapses in the neocortex and GABA_A_-receptor-containing inhibitory postsynapses in the hippocampus, respectively [6]–[8]. Neurexins were shown to be concentrated in presynaptic terminals in the pons and hippocampus [9]. Furthermore, nectins were found to be co-localized with N-cadherin at PAJs, but not at SJs, at mossy fiber-CA3 pyramidal cell synapses in the hippocampus [10]. Nectins comprise a family of four members (nectin-1 nectin-2, nectin-3 and nectin-4) [11], [12]. All members have three immunoglobulin-like loops in their extracellular regions flanked by a single transmembrane region. The cytoplasmic tail of nectins binds the filamentous actin (F-actin)-binding protein, l-afadin and its shorter variant, s-afadin. Although N-cadherin and l-afadin are symmetrically localized at the presynaptic and postsynaptic sides of PAJs at mossy fiber-CA3 pyramidal cell synapses, nectin-1 and nectin-3 are asymmetrically localized at the presynaptic and postsynaptic sides of PAJs, respectively [10].

Afadin is homologous to a human *AF-6* gene product [13], [14]. *AF-6* was originally identified as the fusion partner of the *ALL-1* gene involved in acute myeloid leukemia with chromosome translocation [15]. In mice, rats and humans, afadin is encoded by an *Mllt4* gene that produces several translational products, presumably by alternative splicing. The largest afadin protein, which is called l-afadin, binds F-actin through its F-actin-binding domain in the C-terminus, but other short variants lack this domain. In the brain, l-afadin and s-afadin, the latter of which is one of the short variants, are mainly expressed [13]. Here, l-afadin is referred to simply as afadin. Afadin binds many proteins through multiple domains, including two Ras-associated domains, a forkhead-associated domain, a dilute domain, a PDZ domain, three proline-rich domains, and an F-actin-binding domain from the N-terminus to the C-terminus. The afadin-binding proteins thus far identified include α-catenin, p120^ctn^, ponsin, ADIP, LMO7, PLEKHA7, ZO-1, Rap1, Rit, Rin, Eph receptors, neurexins, Jagged-1, JAM, SPA-1, Bcr, c-Src, LMO2, profilin, and nArgBp2 [14], [16].

We and another group generated the *afadin* straight knockout mouse lines [17], [18]. Because systemic ablation of *afadin* caused early embryonic lethality, an *afadin*-floxed mouse line was generated and camk2a-Cre conditional ablation was utilized to inactivate the *afadin* gene in the hippocampus after postnatal day 9 (P9) [19]. In the mutant mice, the active zone protein, bassoon, and the postsynaptic density protein, PSD-95, accumulate at mossy fiber-CA3 pyramidal cell synapses, but perforated PSDs tend to be more frequently observed than in control mice. Because perforated PSDs are observed in synapses that undergo remodeling [2], these results suggest that afadin is likely to regulate the remodeling of synapses. However, the role of afadin in synaptogenesis remains to be determined.

In the present study, we analyzed the role of afadin in PAJ formation and presynaptic differentiation using a nestin-Cre mouse line, in which the *nestin* promoter starts to operate around E10.5 [20]. These experiments revealed that afadin regulates PAJ formation and presynaptic differentiation at mossy fiber-CA3 pyramidal cell synapses in the stratum lucidum of the hippocampus.

## Materials and Methods

### Mice

The *afadin*-floxed mice [19] and nestin-Cre mice [21] were described previously. The heterozygous mice carrying the *afadin* conditional allele are referred to as *afadin* ^+/f^. Genotyping was performed with a REDExtract-N-Amp Tissue PCR kit (Sigma). The mutant and control samples were prepared from the same litter. The morning after coitus and the day of birth were defined as E0.5 and P0, respectively. All animal experiments were performed in strict accordance with the guidelines of the institution and approved by the administrative panel on laboratory animal care of Kobe University. The protocol was approved by the Committee on the Ethics of Animal Experiments of Kobe University Graduate School of Medicine (Permit Number: P130205). All efforts were made to minimize suffering.

### Western blotting

Mouse forebrains were dissected, placed in tubes, frozen in liquid nitrogen, and stored at −80°C until use. Tissues were homogenized with a Teflon-glass homogenizer in 20 mM Tris-HCl, pH 7.5, 1 mM EDTA, 1 mM Na_3_VO_4_, 10 mM NaF, 1 mM phenylmethylsulfonyl fluoride, 10 µg/ml leupeptin, and 1.5 µg/ml aprotinin. Then, 150 mM NaCl and 10% (wt/vol) glycerol were added to the homogenates. The homogenates were centrifuged at 800×*g* at 4°C for 10 min and the supernatants were collected. Protein concentrations were determined using the Bio-Rad protein assay (Bio-Rad). Protein lysates (20 µg each) were separated by SDS-PAGE, transferred to PVDF membranes, and incubated with antibodies (Abs). Immunodetection was performed with Immobilon Western (Millipore) and a LAS-4000 luminescent image analyzer (Fujifilm).

### Immunohistochemistry and immunocytochemistry

For immunohistochemistry, deeply-anesthetized mice were perfused with an ice-cold fixative composed of 2% paraformaldehyde, 4% sucrose, 1 mM sodium pyruvate, Hanks' balanced salt solution (HBSS) containing 1 mM CaCl_2_ and 1 mM MgCl_2_ (Life Technologies), 3 units/ml heparin sodium, and 10 mM HEPES (pH 7.3). The brains were dissected and incubated in the same fixative at 4°C for 4 h, and then they were dehydrated overnight in 30% sucrose, 1 mM sodium pyruvate, HBSS containing 1 mM CaCl_2_ and 1 mM MgCl_2_, and 10 mM HEPES (pH 7.3). The brains were placed in OCT compound (Tissue Tek) and frozen on dry ice. Sections of 14-µm thickness were mounted on glass slides and incubated at 62°C for 20 min in HistoVT One antigen retrieval solution (Nacalai Tesque), and then blocked at room temperature for 20 min in 100 mM phosphate buffer (PB) (pH 7.4) containing 10% goat serum, 1% bovine serum albumin, and 0.25% Triton X-100. The specimens were incubated at 4°C for 48 h with primary Abs in CanGetSignal immunoreaction enhancer solution B (Toyobo). After washing for 10 min three times in 100 mM PB containing 0.05% saponin, the samples were incubated at 4°C for 24 h with secondary Abs and 1 µg/ml DAPI (Nacalai Tesque) in the immunoreaction enhancer solution. After washing three times for 10 min in 100 mM PB containing 0.05% saponin, the samples were mounted in FluorSave reagent (Merck) and observed with an LSM510 META confocal laser scanning microscope (Carl Zeiss). For immunostaining of cultured hippocampal neurons, cells were fixed with the above-mentioned fixative without heparin sodium at 37°C for 15 min. The fixed cells were permeabilized at room temperature for 5 min with 0.25% Triton-X and 0.005% Tween-20 in Tris-buffered saline (TBS) containing 1 mM CaCl_2_, and then blocked with 10% goat serum in TBS containing 0.005% Tween-20 and 1 mM CaCl_2_ at 37°C for 20 min. Then, the cells were incubated with primary Abs in the solution used for blocking at 4°C overnight. After washing 3 times for 5 min in 0.005% Tween-20 in TBS containing 1 mM CaCl_2_ at room temperature, the cells were incubated with Alexa Fluor-conjugated secondary Abs (Life Technologies) at room temperature for 45 min. Maximum intensity projection images were created from around 10 confocal images collected at a 0.4-µm step along the z-axis with an LSM700 or LSM510 META confocal laser scanning microscope (Carl Zeiss) under exactly the same conditions for both control and afadin-deficient neurons. The immunofluorescence signals for nectin-1, nectin-3, N-cadherin, β-catenin, VGLUT1 and bassoon in cultured neurons were measured in synaptotagmin I –positive punctae that located between 5 µm and 45 µm away from cell bodies along dendrites for each genotype (20 punctae per neuron, totally 100 punctae for each genotype) and subjected to statistical analysis.

### Abs

Rabbit anti-l-afadin and rabbit anti-l/s-afadin were prepared as described [13]. The Abs listed below were purchased from commercial sources: rat anti-nectin-1, clone 48–12 (MBL); rat anti-nectin-3, clone 103-A1 (MBL); mouse anti-N-cadherin, clone 32 (BD Biosciences); rabbit anti-N-cadherin (Takara); rabbit anti-β-catenin (Sigma); rabbit anti-synapsin I (Millipore); guinea pig anti-VGLUT1 (Millipore); mouse anti-bassoon (Enzo Life Sciences); mouse anti-PSD-95, clone 7E3-1B8 (Enzo Life Sciences) and clone K28/43 (NeuroMab); mouse anti-actin (clone C4) (Millipore); and chicken anti-MAP2 (Abcam). Alexa Fluor-conjugated secondary Abs (Life Technologies) were used for immunohistochemistry and immunocytochemistry.

### Cell culture

Hippocampal neuron cultures were prepared from E18.5 embryos, which were generated by the breeding of *afadin* ^f/f^ and *afadin* ^+/f^;nestin-Cre mice. To identify the mutants and littermate controls, the embryos were set aside on ice in 1 mM sodium pyruvate, HBSS without CaCl_2_ and MgCl_2_ (Life Technologies), and 10 mM HEPES (pH 7.3) for approximately 3 h while genotyping. Dissociated hippocampal neurons were prepared as described [22] with slight modifications. In brief, hippocampal neurons dissociated with trypsin were plated at a density of 5–7×10^3^ cells/cm^2^ on poly-L-lysine-coated coverslips in Neurobasal medium (Life Technologies) containing B27 supplement (Life Technologies) and GlutaMAX (Life Technologies), and cultured in a 5% CO_2_ incubator. Neurons for electrophysiology experiments were initially cultured in MEM (Life Technologies) containing 10% fetal bovine serum for 18 h. To label recycling synaptic vesicles, cultured neurons were incubated with the rabbit polyclonal synaptotagmin I luminal domain Ab (Synaptic Systems, #105 102, 1∶50) in culture medium at 37°C for 30 min in 5% CO_2_. To quantify the immunofluorescence signal for synaptotagmin I uptake, the intensities of sixty punctae along dendrites for each genotype were measured and subjected to statistical analysis.

### Electrophysiology

The hippocampal cultures at 14–21 days *in vitro* (DIV) were transferred to a recording chamber and superfused with an external solution (pH 7.4) containing: 148.8 mM Na^+^, 2.7 mM K^+^, 149.2 mM Cl^−^, 2.8 mM Ca^2+^, 2.0 mM Mg^2+^, 11.6 mM HCO_3_^−^, 0.4 mM H_2_PO_4_^−^, 5.6 mM D-glucose, 0.01 mM gabazine, and 10 mg/l phenol red. The cultures were kept at room temperature. Recordings were obtained from the cells held at –70 mV with patch pipettes (2–5 MΩ) using an EPC 10 amplifier (HEKA Elektronik). Pipettes were filled with a solution (pH 7.2) containing 135 mM K-gluconate, 5 mM KCl, 10 mM HEPES, 1 mM EGTA, 2 mM Mg-ATP, 5 mM creatine phosphate, 0.4 mM GTP, and 0.07 mM CaCl_2_. The actual membrane potentials were corrected for the liquid junction potential. Miniature excitatory postsynaptic currents (mEPSCs) were recorded in the presence of tetrodotoxin (1 µM) added to the above-mentioned external solution. mEPSCs were analyzed off-line using Synaptosoft mini analysis software. For recording from synaptically-coupled pairs of neurons, presynaptic action potentials were evoked by injecting depolarizing current (1 ms, 1.5 to 2 nA) at 0.1 Hz. Series resistance (typically between 5 and 15 MΩ) was regularly monitored and cells were excluded if a change of more than 20% occurred. Gabazine, ATP, CrP, EGTA and GTP were purchased from Sigma/Fluka. For data acquisition, signals were filtered at 10 kHz and digitally recorded using PATCHMASTER software (HEKA Elektronik).

### Statistical analysis

Statistical analysis of the difference between mean values was performed with two-tailed Student's *t* test. The criterion for statistical significance was set at *P*<0.05. All values are reported as the mean ± s.e.m.

## Results

### Conditional ablation of *afadin* in the brain

**Figure 1:**
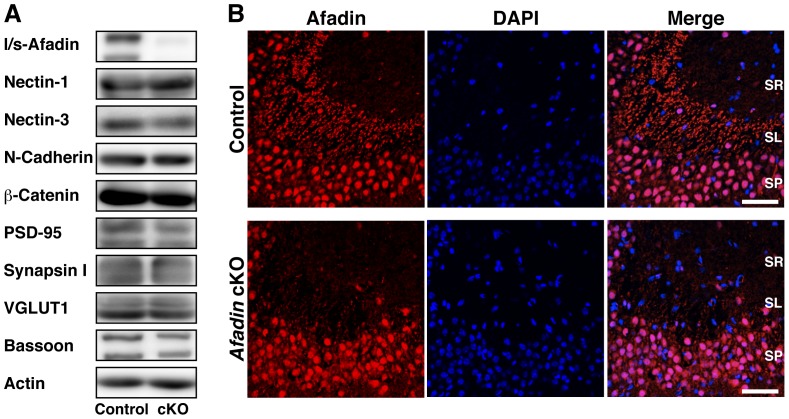
Conditional ablation of *afadin* in the brain. (A) Expression levels of various synaptic components in P14 forebrains. The indicated synaptic proteins were analyzed by Western blotting. Twenty µg of protein lysates were loaded in each lane. (B) Expression pattern of afadin in the CA3 stratum lucidum at P14. Hippocampal sections were stained with the l-afadin Ab (red) and 4′,6-diamidino-2-phenylindole dihydrochloride (DAPI) (blue). The signal for afadin in nuclei was likely non-specific because it was not abolished by genetic ablation of *afadin*. The results shown are representative of three independent experiments. SR, stratum radiatum; SL, stratum lucidum; SP, stratum pyramidale. Control, *afadin* ^+/f^; cKO, *afadin* ^f/f^;nestin-Cre. Scale bars, 25 µm.

In the present study, we used *afadin* ^f/f^;nestin-Cre mice and their *afadin* ^+/f^ littermates as controls. We first confirmed that afadin is indeed ablated in the *afadin* conditional knockout (cKO) brain. Western blotting of P14 brain extracts demonstrated that both the l-afadin and s-afadin proteins were almost lost by the conditional ablation, but the levels of other synaptic components, including nectin-1, nectin-3, N-cadherin, β-catenin, PSD-95, synapsin I, VGLUT1 and bassoon, were largely unaltered (Fig. 1A). These results indicate that afadin expression was specifically ablated in the *afadin* cKO brain.

### Decreased immunofluorescence signals for the PAJ CAMs at mossy fiber-CA3 pyramidal cell synapses in the *afadin* cKO brain

We next examined the role of afadin in the accumulation of nectin-1, nectin-3 and N-cadherin at mossy fiber-CA3 pyramidal cell synapses in the stratum lucidum of the hippocampus. First, we confirmed by immunofluorescence microscopy that afadin expression was ablated in this region in the *afadin* cKO brain (Fig. 1B). The immunofluorescence signal for afadin was observed as dots that aligned along the dendrites of the pyramidal cells at the control CA3 stratum lucidum, as described [10]. In contrast, the signal for afadin was not detected in the same region of the *afadin* cKO brain. These results indicate that the afadin expression was ablated at mossy fiber-CA3 pyramidal cell synapses in the *afadin* cKO brain.

**Figure 2:**
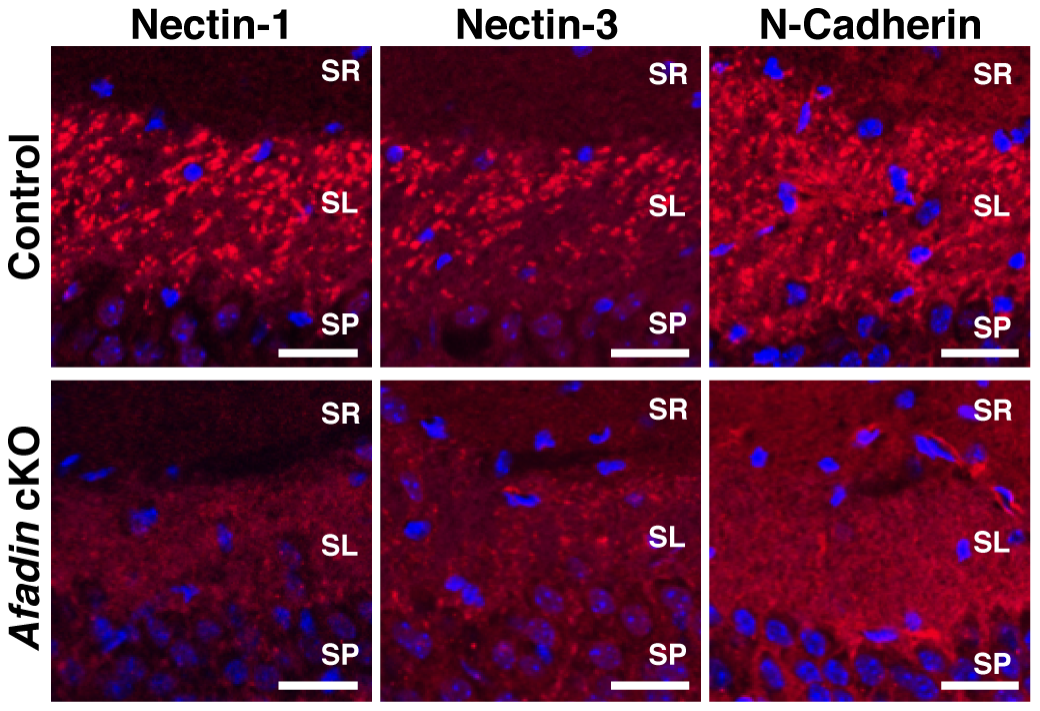
Decreased immunofluorescence signals for the CAMs at the mossy fiber-CA3 pyramidal cell synapses in the *afadin* cKO brain. Coronal hippocampal sections at P14 were stained with the indicated Ab against nectin-1, nectin-3 or N-cadherin (red) and DAPI (blue). The results shown are representative of three independent experiments. SR, stratum radiatum; SL, stratum lucidum; SP, stratum pyramidale. Control, *afadin* ^+/f^; cKO, *afadin* ^f/f^;nestin-Cre. Scale bars, 25 µm.

We next examined the signals for nectin-1, nectin-3 and N-cadherin (Fig. 2), which were observed as dots in the CA3 stratum lucidum of the control hippocampus as previously described [10], [23]. The distributions of these proteins were similar to that of afadin. However, the signals for nectin-1, nectin-3 and N-cadherin disappeared almost completely in the CA3 stratum lucidum of the afadin-deficient hippocampus. These results indicate that afadin is required for the accumulation of the immunofluorescence signals for the CAMs at mossy fiber-CA3 pyramidal cell synapses.

### Decreased immunofluorescence signals for the PAJ CAMs at the synapses in cultured afadin-deficient hippocampal neurons

**Figure 3:**
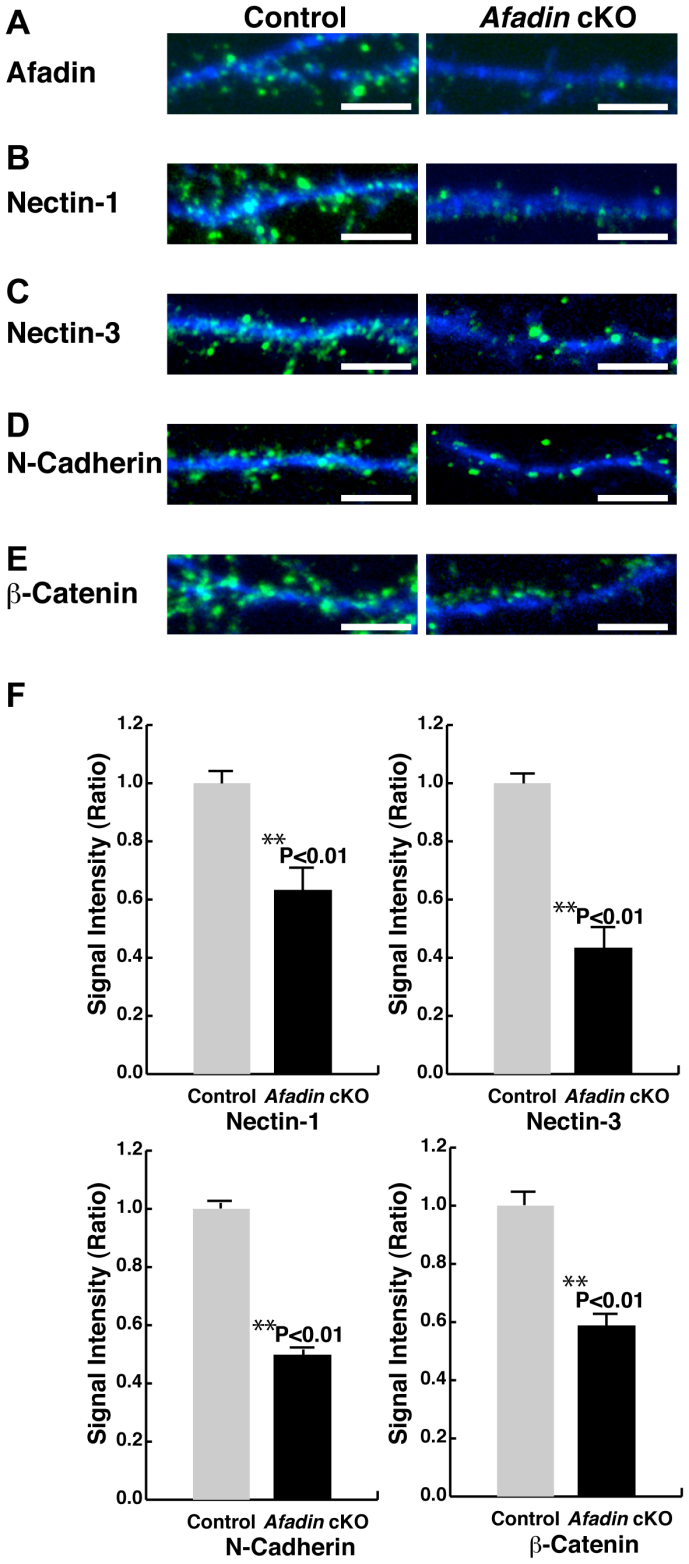
Decreased immunofluorescence signals for the CAMs at the synapses in cultured afadin-deficient hippocampal neurons. (A–E) Expression patterns of afadin, nectin-1, nectin-3, N-cadherin and β-catenin in the cultured hippocampal neurons. The cultured neurons at 14 DIV were double-stained with the MAP2 Ab and the indicated Ab against afadin, nectin-1, nectin-3, N-cadherin or β-catenin. The results shown are representative of three independent experiments. Scale bars, 5 µm. (F) Ratio of the intensity of punctae positive for nectin-1, nectin-3, N-cadherin or β-catenin in the afadin-deficient neurons relative to control neurons. Punctae that located between 5 µm and 45 µm away from cell bodies along dendrites were analyzed (100 punctae from five neurons were analyzed for each genotype). Error bar, s.e.m. Control, *afadin* ^+/f^; cKO, *afadin* ^f/f^;nestin-Cre.

To confirm the role of afadin in the accumulation of the immunofluorescence signals for nectin-1, nectin-3 and N-cadherin at the synapses *in vitro*, hippocampal neuron cultures were prepared from the *afadin* ^f/f^;nestin-Cre embryos and control *afadin^+^* ^/f^ embryos. We first confirmed that afadin was ablated in the hippocampal neurons from the *afadin* ^f/f^;nestin-Cre embryos. The immunofluorescence signal for afadin was observed as dots along dendrites in the control neurons at 14 DIV (Fig. 3A). However, in the afadin-deficient neurons, the signal for afadin was not observed. These results indicate that afadin expression was indeed ablated in the cultured hippocampal neurons from the *afadin* ^f/f^;nestin-Cre embryos.

We then examined the effects of *afadin* ablation on the accumulation of nectin-1, nectin-3, N-cadherin and β-catenin in the cultured hippocampal neurons. In the control neurons, the signals for nectin-1, nectin-3, N-cadherin and β-catenin were observed as dots along the dendrites at 14 DIV (Fig. 3B–F). However, in the afadin-deficient neurons, the signals for nectin-1, nectin-3, N-cadherin and β-catenin decreased significantly. These results indicate that afadin is required for the accumulation of the immunofluorescence signals for the CAMs and β-catenin at the synapses in cultured hippocampal neurons.

### Decreased immunofluorescence signals for the presynaptic components at mossy fiber-CA3 pyramidal cell synapses of the *afadin* cKO brain

**Figure 4:**
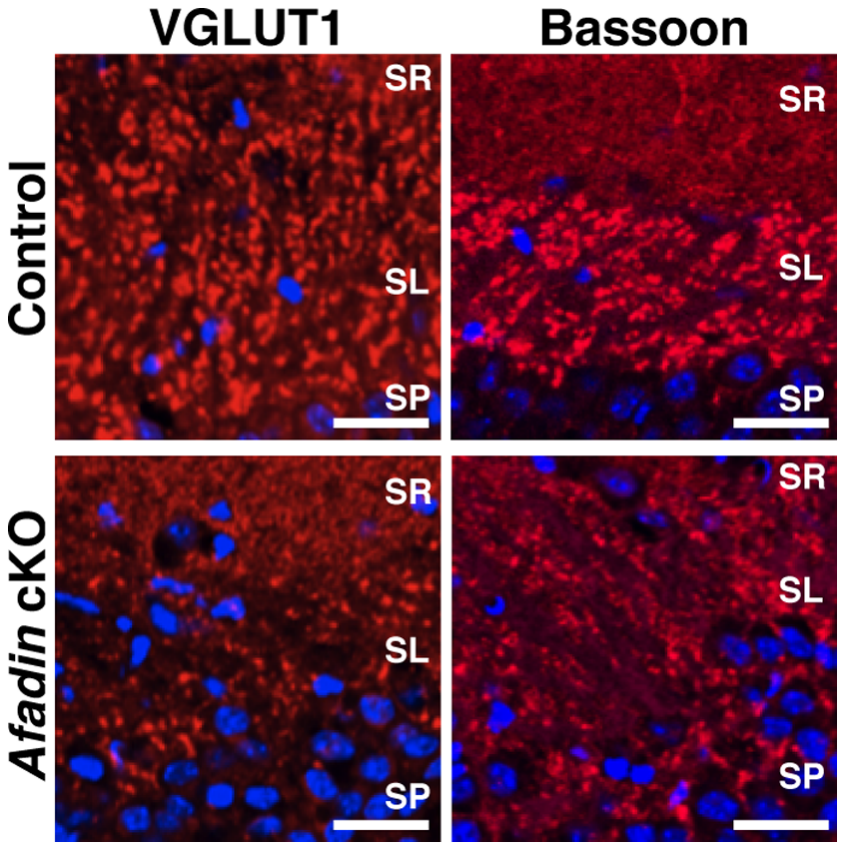
Decreased immunofluorescence signals for the presynaptic components at the mossy fiber-CA3 pyramidal cell synapses of the *afadin* cKO brain. Coronal hippocampal sections at P14 were stained with the indicated Ab against VGLUT1 or bassoon (red) and DAPI (blue). The results shown are representative of three independent experiments. SR, stratum radiatum; SL, stratum lucidum; SP, stratum pyramidale. Control, *afadin* ^+/f^; cKO, *afadin* ^f/f^;nestin-Cre. Scale bars, 25 µm.

We next examined the role of afadin in the accumulation of the immunofluorescence signals for the presynaptic components, VGLUT1 and bassoon, at mossy fiber-CA3 pyramidal cell synapses. VGLUT1 is a component of synaptic vesicles [24] and bassoon is a component of the active zone [25]. The immunofluorescence signals for VGLUT1 and bassoon were observed as dots in the CA3 stratum lucidum of the control hippocampus (Fig. 4). However, the signals for these proteins were markedly decreased, but not abolished, in this region of the afadin-deficient hippocampus. These results indicate that afadin is required for the accumulation of the immunofluorescence signals for the presynaptic components at a considerable population of mossy fiber-CA3 pyramidal cell synapses.

### Decreased immunofluorescence signals for the presynaptic components at synapses in cultured afadin-deficient hippocampal neurons

**Figure 5:**
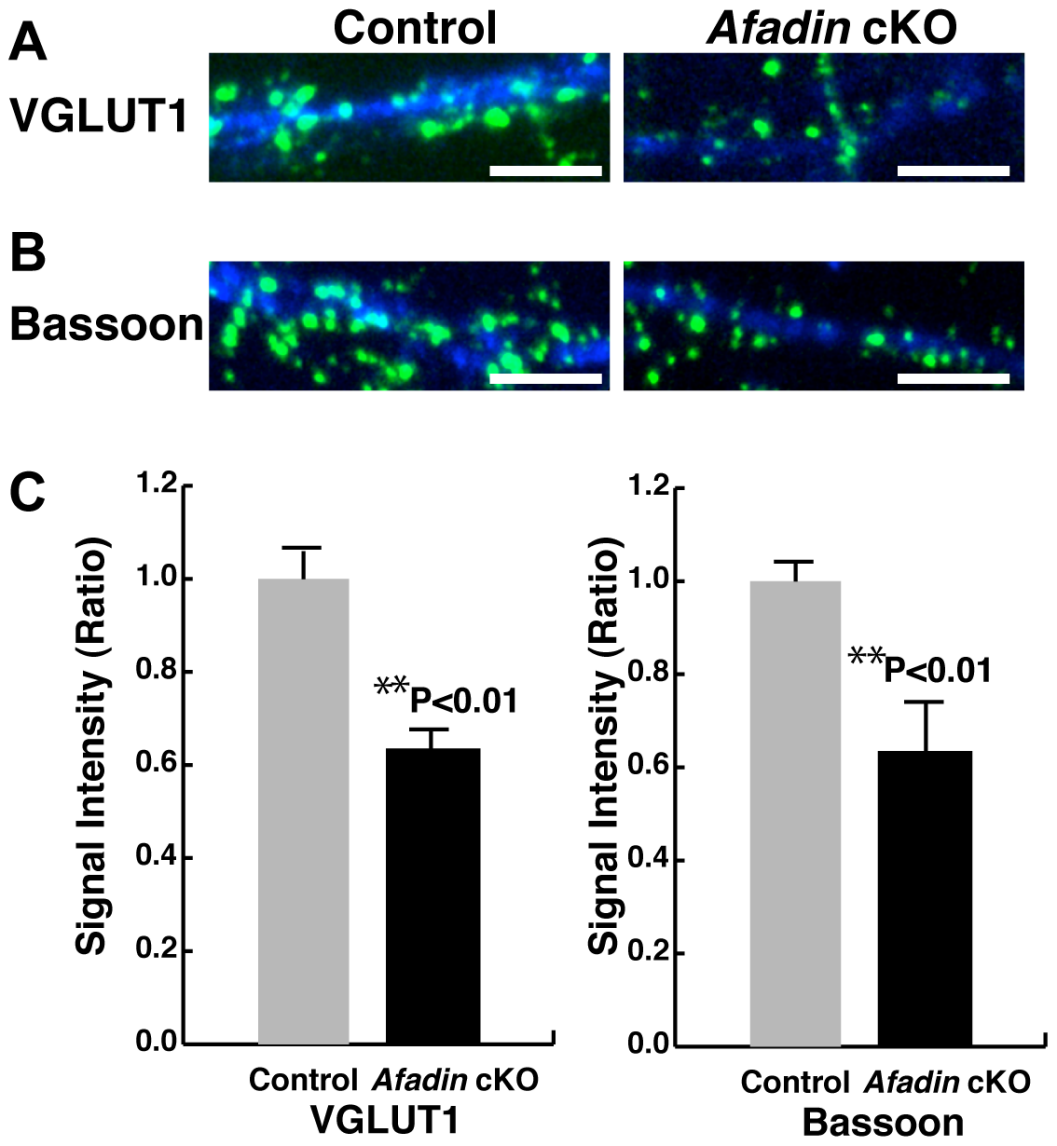
Decreased immunofluorescence signals for the presynaptic components at the synapses in cultured afadin-deficient hippocampal neurons. (A, B) Expression patterns of VGLUT1 and bassoon in the cultured hippocampal neurons. The cultured hippocampal neurons at 14 DIV were double-stained with the MAP2 Ab and the indicated Ab against VGLUT1 or bassoon. The results shown are representative of three independent experiments. Scale bars, 5 µm. (C) Ratio of the intensity of punctae positive for VGLUT1 or bassoon in the afadin-deficient neurons relative to control neurons. Punctae that located between 5 µm and 45 µm away from cell bodies along dendrites were analyzed (100 punctae from five neurons were analyzed for each genotype). Error bar, s.e.m. Control, *afadin* ^+/f^; cKO, *afadin* ^f/f^;nestin-Cre.

Next, we confirmed the role of afadin in the accumulation of the immunofluorescence signals for the presynaptic components at synapses in the cultured hippocampal neurons. The immunofluorescence signals for VGLUT1 and bassoon were observed as dots along the dendrites in control neurons at 14 DIV (Fig. 5). However, in the afadin-deficient neurons, the signals for these proteins were decreased significantly but not abolished. These results indicate that afadin is required for the accumulation of the immunofluorescence signals for the presynaptic components at synapses in a considerable population of cultured hippocampal neurons.

### Decrease in functional synapses in cultured afadin-deficient hippocampal neurons

**Figure 6:**
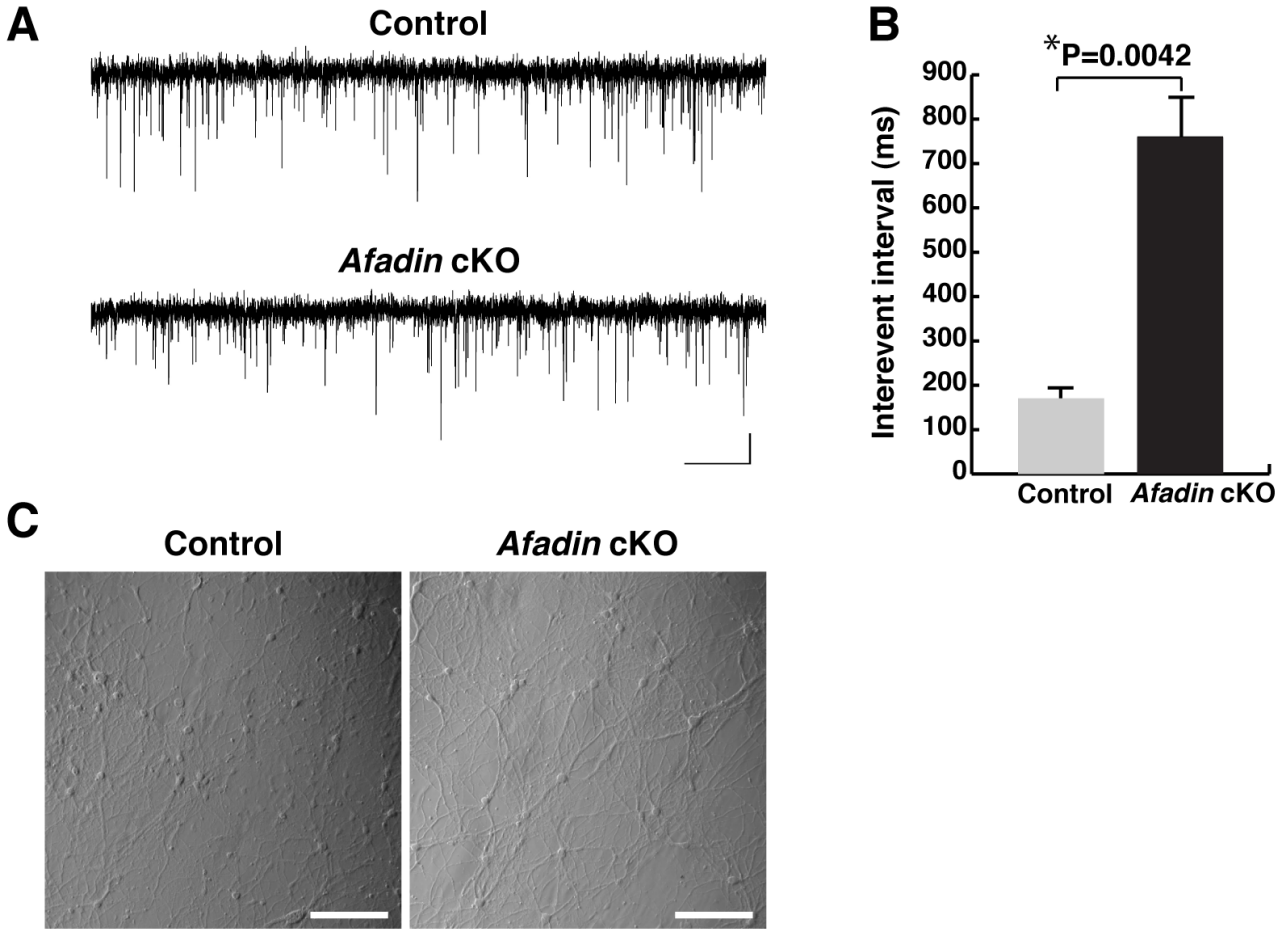
Decreased functional synapses in cultured afadin-deficient hippocampal neurons. (A) Example mEPSC traces. The vertical and horizontal bars denote 5 pA and 1 s, respectively. (B) The average interevent interval of mEPSCs (n = 12 in both the control and *afadin* cKO neurons). (C) Appearance of the cultured neurons used for analysis of mEPSCs. The neuron density is similar between control and *afadin* cKO neurons. Scale bars, 200 µm. Control, *afadin* ^+/f^; cKO, *afadin* ^f/f^;nestin-Cre.

Thus far, we have shown that the immunofluorescence signals for the PAJ CAMs mostly disappeared in mossy fiber-CA3 pyramidal cell synapses in the stratum lucidum of the the *afadin* cKO hippocampus and cultured afadin-deficient hippocampal neurons, while the signals for the presynaptic markers decreased less extensively. From these results, we could not distinguish between two possible mechanisms: 1) afadin is required for PAJ formation and presynaptic differentiation; 2) afadin is required for the accumulation of the presynaptic marker proteins at developing synapses. To address these issues, we recorded mEPSCs in cultured hippocampal neurons. We detected mEPSCs in both the control and afadin-deficient neurons, but the interevent interval was prolonged in the afadin-deficient neurons compared with control neurons (Fig. 6). These results indicate that presynaptic functions were suppressed or a number of synapse was reduced in the afadin-deficient neurons. Therefore, it is likely that afadin is at least required for PAJ formation and presynaptic differentiation in most mossy fiber-CA3 pyramidal cell synapses.

### Impaired presynaptic functions in cultured afadin-deficient hippocampal neurons

**Figure 7:**
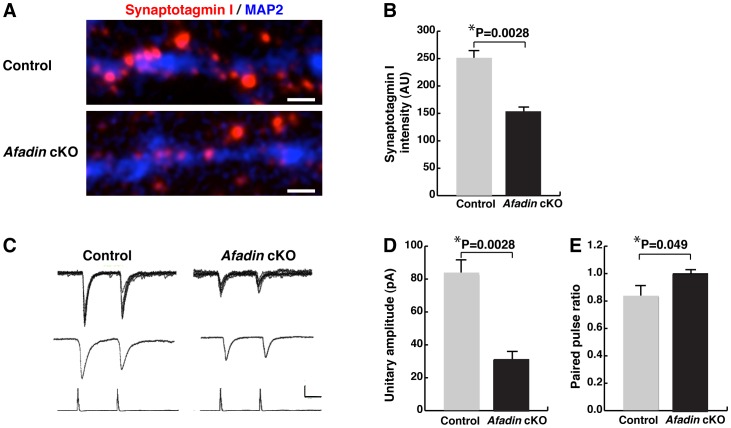
Impaired presynaptic functions in cultured afadin-deficient hippocampal neurons. (A, B) Labeling of functional synapses in cultured live hippocampal neurons. (A) Recycling synaptic vesicles labeled by incubation of cultured live hippocampal neurons at 14 DIV with the synaptotagmin I luminal domain Ab. Scale bars, 1 µm. (B) Quantification of the total integrated intensity of the internalized synaptotagmin I Ab. Sixty punctae for each genotype were analyzed. (C–E) Modulation of presynaptic vesicular release by afadin in cultured hippocampal neurons. (C) Examples of postsynaptic responses evoked by paired action potentials in a presynaptic cell with a 50-ms interval in control and *afadin* cKO neurons. Superimposed images for 15 recordings of unitary EPSC (top), average unitary EPSC (middle), and action potentials (bottom). A vertical scale bar denotes 20 pA for the top and middle rows and 5 mV for the bottom row; a horizontal scale bar denotes 20 ms. (D) The average unitary amplitudes (n = 10 and 12 in control and *afadin* cKO neurons, respectively). (E) paired-pulse ratios (n = 8 and 11 in control and *afadin* cKO neurons, respectively). Error bars, s.e.m. Control, *afadin* ^+/f^; cKO, *afadin* ^f/f^;nestin-Cre.

In the last set of experiments, we measured vesicle recycling and performed paired recordings to ask whether presynaptic functions were impaired in cultured afadin-deficient hippocampal neurons. Vesicle recycling was assessed by measuring synaptotagmin I uptake in the cultured hippocampal neurons. The vesicle-recycling rate per synapse was markedly decreased in the synapses of the afadin-deficient hippocampal neurons compared with control neurons, indicating that presynaptic functions were impaired in the mutant neurons (Fig. 7A, B). Analysis of paired recordings demonstrated that the amplitude of the unitary EPSC was smaller in the cultured afadin-deficient neurons than in the control neurons (Fig. 7C, D). Moreover, an increased paired-pulse ratio was observed in the cultured afadin-deficient neurons, suggesting a decreased release probability in these neurons (Fig. 7C, E). These results indicate that afadin is also required for presynaptic functions in cultured hippocampal neurons.

## Discussion

Here, we showed that the genetic ablation of *afadin* using nestin-Cre mice severely decreased the accumulation of the immunofluorescence signals for PAJ components at mossy fiber-CA3 pyramidal cell synapses in the stratum lucidum of the hippocampus. In addition, we confirmed the similar effects of afadin in cultured hippocampal neurons and further showed that the ablation of *afadin* impaired presynaptic functions. These results indicate that afadin is required for PAJ formation in almost all mossy fiber-CA3 pyramidal cell synapses.

Afadin is ubiquitously expressed and consists of multiple domains, and no structurally-related homologous proteins have been identified, suggesting that it plays pivotal roles in multiple biological processes [13]. In fact, afadin deficiency leads to the disruption of cell-cell junctions and cell polarity in neuroepithelial cells and causes early embryonic lethality [17], [18]. We previously showed that nectins first form cell–cell adhesions and then recruit cadherins to the nectin-based cell–cell adhesion sites through afadin, resulting in the formation of AJs, in both fibroblasts and epithelial cells [12]. Nectins bind afadin, which in turn binds α-catenin associated with cadherins through β-catenin. These molecular linkages are required for the association of cadherins with nectins. Therefore, it is likely that afadin cooperatively functions with nectins and cadherins to form PAJs in mossy fiber-CA3 pyramidal cell synapses.

The major features of presynaptic differentiation include the formation of active zones and the accumulation of synaptic vesicles in the presynaptic side of synapses. We showed here that the genetic ablation of *afadin* considerably decreased the accumulation of the immunofluorescence signals for the active zone component, bassoon, and the synaptic vesicle component, VGLUT1, at mossy fiber-CA3 pyramidal cell synapses and synapses in cultured hippocampal neurons. These results indicate that the active zones are formed in an afadin-dependent manner in most mossy fiber-CA3 pyramidal cell synapses in which PAJs are formed, while they are also formed in an afadin-independent manner in a considerable population of these synapses in which PAJs might not be formed. Because the residual synapses that were formed in afadin-deficient neurons showed impaired recycling of synaptic vesicles and abnormal paired recordings, the active zones formed in an afadin-independent manner may not be fully differentiated in their structures and functions. Other CAMs such as neuroligins may be involved in the afadin-independent formation of the active zones at mossy fiber-CA3 pyramidal cell synapses, but it is currently unknown whether the active zones are formed in afadin-dependent and -independent manners in the same or different synapses, how the active zones are formed in afadin-dependent and -independent manners, or whether PAJs are formed in advance and then the active zones are formed or vice versa.

We previously showed that the immunofluorescence signals for nectin-1 and nectin-3 were almost completely lost in mossy fiber-CA3 pyramidal cell synapses in the *afadin* ^f/f^;camk2a-Cre hippocampus in which the *afadin* gene was inactivated in excitatory neurons after synaptogenesis, and that the signals for N-cadherin and β-catenin were also almost completely lost [19]. However, the immunofluorescence signal for bassoon and PSD-95 were not altered there in the *afadin* ^f/f^;camk2a-Cre hippocampus. These earlier observations together with the present results indicate that afadin is required for PAJ formation and presynaptic differentiation in mossy fiber-CA3 pyramidal cell synapses, but is not required to maintain the accumulation of these components after the synapses are formed. Interestingly, *MLLT4/AFADIN* expression was reduced in the postmortem schizophrenic brain [26]. In addition, genetic ablation of *nectin-1* or *nectin-3* decreased PAJ numbers in mossy fiber-CA3 pyramidal cell synapses [23]. Furthermore, mutations in *NECTIN-1* were shown to be a cause of mental retardation in humans [27]. Taken together, these results indicate the importance of afadin and its binding proteins, nectin-1 and nectin-3, in both physiology and pathology.

It was shown that AF-6/afadin regulates the morphology of dendritic spines in cultured hippocampal neurons [28]. Additionally, it was shown that afadin is required for the maintenance of dendritic arborization and synapse number in cultured hippocampal neurons [29]. These two studies employed afadin knockdown or overexpression of mutant forms of afadin. These earlier observations indicate that afadin regulates synaptogenesis through postsynaptic differentiation. Taken together with the present results, it is likely that afadin is involved in synaptogenesis through both presynaptic and postsynaptic differentiation. In contrast, it was recently shown by use of conditional *afadin*-deficient mice with a Nex-Cre mouse line that the genetic ablation of *afadin* neither changed the localizations of nectin-1 and nectin-3 in the CA1 region nor perturbed the presynaptic functions in the CA3 region, although it was shown that synapse number was decreased in the CA1 region of the afadin-deficient hippocampus [30]. Some of these results are apparently inconsistent with our present results. The exact reason for this inconsistency is not known, but it may be due to the different regions analyzed in the hippocampus, the different promoters used for the Cre mice, the different nectin Abs used, or the different samples used for electrophysiology experiments.
